# Angiotensin II Increased Neuronal Stem Cell Proliferation: Role of AT2R

**DOI:** 10.1371/journal.pone.0063488

**Published:** 2013-05-15

**Authors:** Jie Chao, Lu Yang, Shilpa Buch, Lie Gao

**Affiliations:** 1 Department of Cellular and Integrative Physiology, University of Nebraska Medical Center, Omaha, Nebraska, United States of America; 2 Department of Pharmacology and Experimental Neuroscience, University of Nebraska Medical Center, Omaha, Nebraska, United States of America; Rutgers University, United States of America

## Abstract

Angiotensin II (Ang II), known a potent vasoactive substance in the renin-angiotensin system in the brain, plays a critical role in systemic blood pressure control. However, increasing evidence indicated that the physiological role of Ang II go beyond its vasoactive effect. In the present study, we demonstrated that Ang II type-1 receptor (AT1R) and type-2 receptor (AT2R) were expressed in primary rat hippocampal neuronal stem cells (NSCs). Treatment of rat hippocampal NSCs with Ang II increased cell proliferation. Pretreatment of NSCs with specific AT2R, but not AT1R, antagonist significantly suppressed Ang II-induced cell proliferation. Furthermore, Ang II stimulated ERK and Akt phosphorylation in NSCs. Pretreatment of MEK inhibitor, but not PI3K inhibitor, inhibited Ang II-induced ERK phosphorylation as well as cell proliferation. In addition, stimulation of NSCs with Ang II decreased expression of K_V_ 1.2/K_V_ 3.1 channels and blocked K^+^ currents which lie downstream of ERK activation. Taken together, these findings underpin the role of AT2R as a novel target that regulates cell proliferation mediated by Ang II with implications for therapeutic intervention for regulation of neurogenesis.

## Introduction

It is well known that Angiotensin II (Ang II) is the principal vasoactive substance of the renin-angiotensin-system (RAS) with a variety of physiological actions, including vasoconstriction, aldosterone release, and cell growth [1], [2]. Moreover, increasing evidence highlighted that brain Ang II plays an important role in mediating diverse function such as neuronal injury, neuroinflammation and cognitive function via a brain-specific RAS [3], [4], [5], [6]. Ang II exerts its biological effect via binding two major receptors, the Ang II type-1 receptor (AT1R) and type-2 receptor (AT2R). Although previous study reported that AT2R is predominantly expressed in the fetus and its densities decline rapidly after birth [7], our recent findings demonstrated that in both rats and mice, AT2R expression in the brain is lower in fetus and neonate than that in adults, and that AT1R exhibits an opposite expression profile [8], suggesting an involvement of a potentially important functional role for AT1R and AT2R in brain developmental processes such as neurogenesis.

It is now well-known that neurogenesis is persistent in mammalian brain until adult [9], which is critical for specific cognitive functions, such as learning & memory [10], [11]. Active neurogenesis occurs throughout life and relies upon the proliferation, migration and proper differentiation of NSCs and is regulated by a variety of physiological and pathological stimuli [12], [13], [14]. Despite significant progress has been made in NSC proliferation, the role of Ang II in rat hippocampus NSC proliferation has never been investigated.

Ang II has been clearly demonstrated to induce cell proliferation [15], [16]. It has been postulated that the AT1R and AT2R have opposing actions on proliferation, although this issue remains controversial. Majority of Ang II-mediated cell proliferation has been attributed to AT1R as evidenced that many studies have more precisely investigated the role of AT1R in cell proliferation [15], [16], [17]. On the other hand, accumulating evidence indicated that activation of AT2R promoted neuronal differentiation [18], [19], [20] and neurite outgrowth [21], [22]. Controversially, despite previous study demonstrated that stimulation of AT2R inhibited cell proliferation in PC12W cells [18], other studies have suggested an association of AT2R with cell proliferation [23], [24], [25]. Whether the effect of Ang II on NSC proliferation is mediated by the AT1R or the AT2R remains unknown.

It has been demonstrated that voltage-gated potassium channels (K_V_) affect cell mitogenesis, which was initially reported in human T lymphocytes [26]. Recent studies suggest that K_V_ play an important role in controlling proliferation in numerous types of cell, including glial cells, lymphocyte, endothelial cells, bereast and prostate cancer cells, and stem cell [27], [28], [29], [30], [31]. Several types of K^+^ current have been found in NSCs, such as IK_DR_ (encoded by K_V_ 1.2, K_V_ 1.5 and K_V_ 1.6), I_A_ (encoded by K_V_ 1.4), TEA-sensitive IK_DR_ (encoded by K_V_ 3.1) [32], [33], [34], in which, blockage of K_V_ 1.3 and K_V_ 3.1 channels has been reported to increase NSC proliferation [35]. Interestingly, Ang II has been shown to regulate both K^+^ current and K^+^ channel expression in different types of cell, including rostral ventrolateral medulla neurons [36], mesenchymal stem cells [37]. For instance, Ang II via AT1R increases neuronal firing rate and reduces K_V_ and A-type K^+^ currents [38], [39]. AT2R also can modulate K^+^ currents in cultured neurons [40] and ventricular myocytes [41]. However, it remains to be clarified whether K_V_ channels modulate Ang II-mediated proliferation in NSCs and, if so, AT1R or AT2R is involved in regulation of K^+^ currents.

In the current study, we have investigated the effect of Ang II, which is the terminal effector arm of RAS, on NSC proliferation. We show direct evidence that Ang II/AT2R axis in NSCs may contribute to the NSC proliferation via a previously unidentified role of AT2R. Moreover, we have also demonstrated that the enhancement of Ang II in the process of NSC proliferation is mainly mediated by ERK signaling with downstream blockage of K^+^ channel.

## Materials and Methods

### Reagents

Ang II, PD123319, losartan, CGP42112A, tetraethylammonium chloride (TEA), α-Dendrotoxin (α-DTX), 4-Aminopyridine (4-AP) and tetrodotoxin (TTX) were purchased from Sigma Chemicals (St. Louis, MO). The specific MEK1/2 inhibitor U0126 and PI3K inhibitor LY294002 was purchased from Calbiochem (San Diego, CA).

### Animals

Pregnant female Sprague Dawley rats were purchased from Charles River Laboratories, Inc. (Wilmington, MA). All of animals were housed under conditions of constant temperature and humidity on a 12-h light, 12-h dark cycle, with lights on at 0700 h. Food and water were available ad libitum. All animal procedures were performed according to the protocols approved by the Institutional Animal Care and Use Committee of the University of Nebraska Medical Center.

### Isolation, Differentiation & Characterization of NSCs

NSCs derived from the hippocampus of embryonic day18 (E18) fetus were cultured in substrate-free tissue culture T75 flasks as reported by Tian e*t* *al* [42]. After 4–7 days, NSCs formed neurospheres and were dissociated with Trypsin-EDTA for 20 min at 37°C and plated on poly-D-lysine pre-coated plates (density: 1×10^5^ cells/well for 24-well plate, 1×10^4^ cells/well for 96-well plate) and were used if found more than 90% nestin+ (a marker for progenitor cells).

### Western Blotting

NSCs were lysed using the Mammalian Cell Lysis kit (Sigma, St. Louis, MO). Cell lysis containing 20 µg of protein were subjected to electrophoresis on a 12% sodium dodecyl sulfate-ployacrylaminde (SDS) gel and blotted onto a polyvinylidene difluoride membrane with a semi-dry blotting apparatus. After blocking, membranes were then probed with antibodies recognizing the AT1R (1∶250, Santa Cruz), AT2R (1∶250, Abcam), p-ERK (1∶500, Cell Signaling, Danvers), p-Akt (1∶500, Cell Signaling, Danvers) and β-actin (1∶5000, Santa Cruz). Secondary antibodies were alkaline phosphatase conjugated to goat anti mouse/rabbit IgG (1∶5000). Signals were detected by chemiluminescence (Pierce, Rockford, IL). All of the Western blot experiments were repeated three times individually and representative blots are presented in the figures.

### Immunocytochemistry

Dissociated NSCs were plated on the coverslip. The cells at different time were fixed with 4% paraformaldehyde for 20 minutes at room temperature according to the experiment protocol. The cells were permeabilized and blocked with solution containing 10% normal goat serum (NGS), 0.3%Triton X-100 in PBS at room temperature for 2 hours. Next, the cells were incubated with primary antibody (mouse anti-Nestin: 1∶250, Millipore; goat anti-AT1R: 1∶250, Santa Cruz; rabbit anti-AT2R, 1∶250, Abcam) in 10% NGS, 0.3%Triton X-100 in PBS at 4°C overnight. Following 3 washes with PBS, the cells were exposed to secondary fluorescence antibody for 2 hr. After washing with PBS for 3 times, the cells were mounted on the slide with anti-fade reagent with DAPI at room temperature. The slides were examined with a laser confocal microscope (Leica TSC STED).

### Quantitative Real-Time PCR

RNA was extracted using TRIZOL Reagent (Invitrogen, NY) according to the manufacturer’s instructions. The RNA quality and quantity was verified using NanoDrop 2000 (Thermo, MI). Total RNA was reverse-transcribed using the iScript cDNA synthesis kits (Bio-RAD, CA) according to manufacturer’s protocols. Quantitative RT-PCR assays were carried out using SsoFast Eva Green Supermix RT-PCR kit (Bio-RAD, CA). Relative quantification was analyzed using PTC-200 (Bio-RAD, CA) and Chromo 4 continuous fluorescence detector (Bio-RAD, CA). In each experiment, GAPDH RNA was amplified as a reference standard. Expression data for different K_V_ channel after Ang II treatment was first normalized against GAPDH RNA as ΔCT, and then the relative expression was compared to untreated group using the ΔΔCT method for quantification in Opticon Monitor software (Bio-RAD, CA). Relative fold changes were determined as RQ values. The primers were designed using online software at https://www.genscript.com/ssl-bin/app/primer and synthesized in the Eppley Cancer Institute Molecular Biology Core Laboratory on the campus of the University of Nebraska Medical Center.

### Whole Cell Patch-clamp to Measure Potassium Current

The whole cell patch-clamp technique was used to determine the effect of Ang II on K^+^ currents in NSCs. This experiment was carried out using an Axopatch 200 A amplifier (Axon Instruments, Foster City, CA). Measurements of the K^+^ current were performed in episodic-stimulation mode. For the K^+^ current measurement, patch pipettes were filled with (in mM) 135 KCl, 5 EGTA, 10 HEPES, 2 MgCl_2_, 0.25 CaCl_2_, 1 ATP, 0.1 GTP and 15 glucose, pH 7.2. The extracellular solution consisted of the following composition (in mM): 134 NaCl, 5.4 KCl, 2 MgCl_2_, 10 HEPES, 10 glucose, 1.35 CaCl_2_, 0.3 NaH_2_PO_4_ and 0.3 CdCl_2_, pH 7.4. Na^+^ channels were blocked by TTX (3 µM). Cell membrane capacitance was determined by integrating the capacitive current evoked by a voltage step from 0 to 5 mV and dividing the resulting charge by the voltage step. Currents were not leak subtracted. Current traces were sampled at 10 kHz and filtered at 5 kHz. Holding potential was −80 mV. Current-voltage relations were elicited by test potentials over the range of −80 mV to +80 mV, with duration of 400 ms in 20 mV increments (5 s between steps). Peak currents were measured for each test potential.

### Cell Proliferation Assay

Cell proliferation was measured by CyQUANT® NF Cell Proliferation Assay Kit (Invitrogen, NY). Briefly, NSCs dissociated from neurosphere were seeded in 96-well plates at a density of 10^4^ cells/well for 2 days and were pre-treated with AT1R antagonist-Losartan, AT2R antagonists-PD123319, MEK inhibitor-U0126 or PI3K inhibitor-LY294002 for 1 hr followed by subsequent treatment with Ang II for 48 hrs. Then, 200 µl of the CyQUANT® GR dye/cell-lysis buffer was added into each well and incubated in the CO_2_ incubator for 15 min. Without washing, fluorescence intensity of each well was obtained using a Dynatech MR5000 plate counter at excitation and emission wavelengths of 480 and 520 nm, respectively.

### Viral Transduction in NSCs

NSCs dissociated from neurosphere were seeded in 24-well plates at a density of 10^5^ cells/well. At this time, Ad5-SYN-AT2R-IRES-EGFP or Ad5-SYN-EGFP were added to the culture medium (5×10^7^ infectious units per well), three days later followed by monitoring for AT2R expression using western-blot analyses based upon previous report [43], and performed the cell proliferation experiments.

### Statistical Analysis

Data were expressed as mean ± SEM. Significance of differences between control and samples treated with various drugs was determined by one-way ANOVA followed by post hoc least significant difference (LSD) test. Values of P<0.05 were considered as statistically significant.

## Results

### Ang II Increased Cell Proliferation of NSCs through AT2R

**Figure 1:**
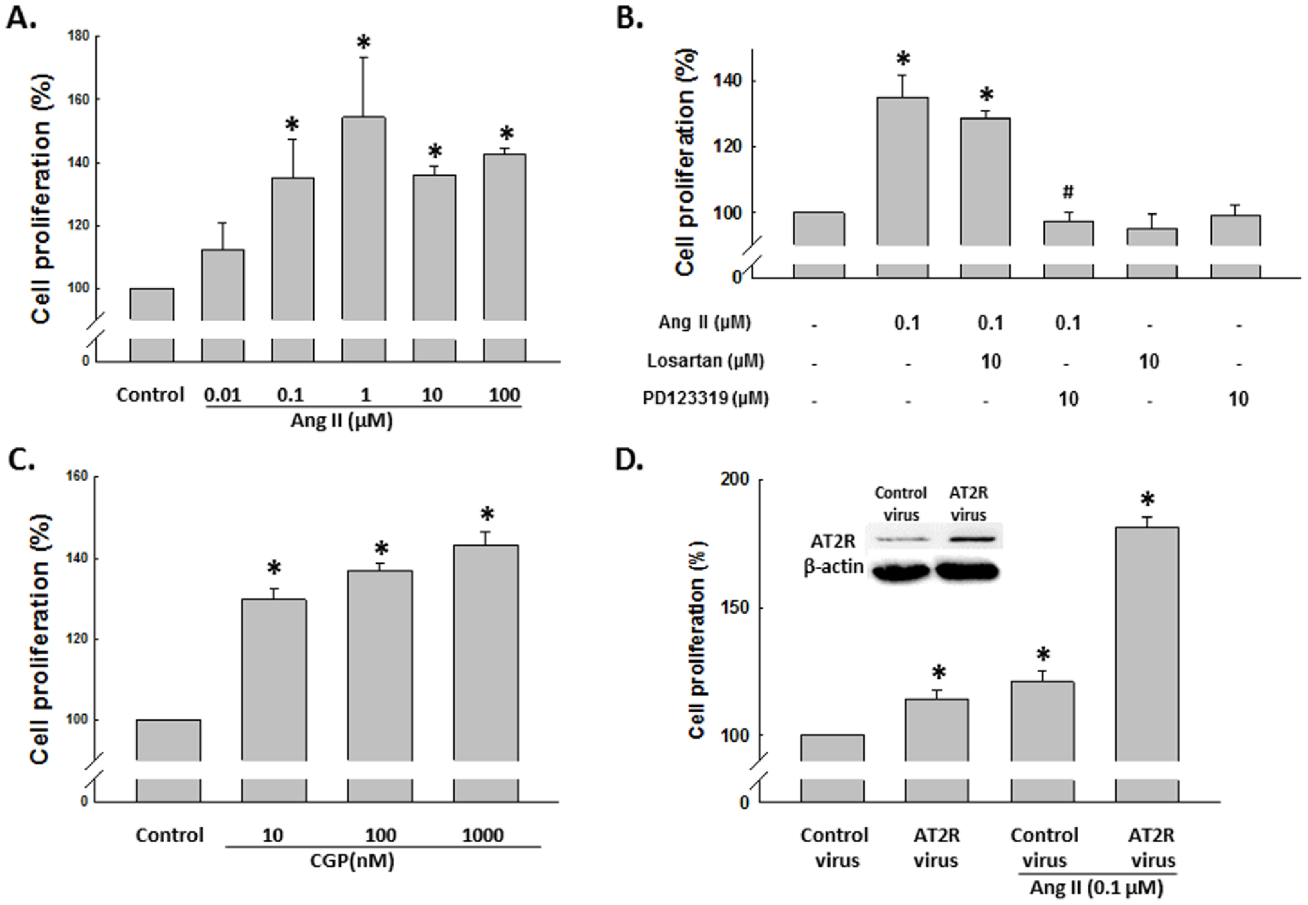
Effect of Ang II on NSC proliferation. A) Ang II (0.01, 0.1, 1, 10 and 100 µM) increased NSC proliferation in a concentration-dependent manner. NSCs were treated with different concentrations of Ang II for 48 hours followed by CyQUANT assay. B) The blockade of AT1R and AT2R on NSCs proliferation. Cells were pretreated with specific antagonist to AT1R or AT2R for 1 hour followed by treatment with Ang II for another 48 hours. Cell proliferation was assessed by CyQUANT assay. C) CGP42112A (10, 100 and 1000 nM) induced increase of NSC proliferation in a concentration-dependent manner. NSCs were treated with different concentrations of CGP42112A for 48 hours followed by CyQuant assay. D) Effect of AT2R overexpression on NSC proliferation. AT2R expression in NSCs transduced with control virus and AT2R virus for 72 hours as shown in the inset. *P<0.05 *vs* control, ^#^P<0.05 *vs* Ang II-treated group, n = 5 in each group.

To assess the effects of Ang II on the cell proliferation of NSCs, rat NSCs were exposed to varying concentrations of Ang II for 48 h and cell proliferation assessed by CyQuant assay. As shown in Figure 1A, Ang II induced NSC proliferation in a concentration-dependent manner. Increasing concentrations of Ang II (0.01, 0.1, 1, 10, 100 µmol/L) resulted in increased cell proliferation by 12%, 35%, 54%, 36% and 43%, respectively. We next assessed whether the effect of Ang II was mediated via its binding to its cognate receptor AT1R and AT2R. As shown in Figure 1B, AT2R selective antagonist-PD123319, but not AT1R selective antagonist-losartan, blocked cell proliferation induced by Ang II in rat NSC culture. To further consolidate these observations, we treated NSCs with AT2R agonist-CGP42112A and found that CGP42112A increased cell proliferation, indicating that the effect of Ang II on cell proliferation is mainly through AT2R pathway (Figure 1C). Moreover, as shown in Figure 1D, NSCs with overexpression of AT2R showed higher response in proliferation than that of control group, further confirming AT2R is critical for Ang II-mediated cell proliferation.

### Expression Pattern of AT1R and AT2R in NSCs

**Figure 2:**
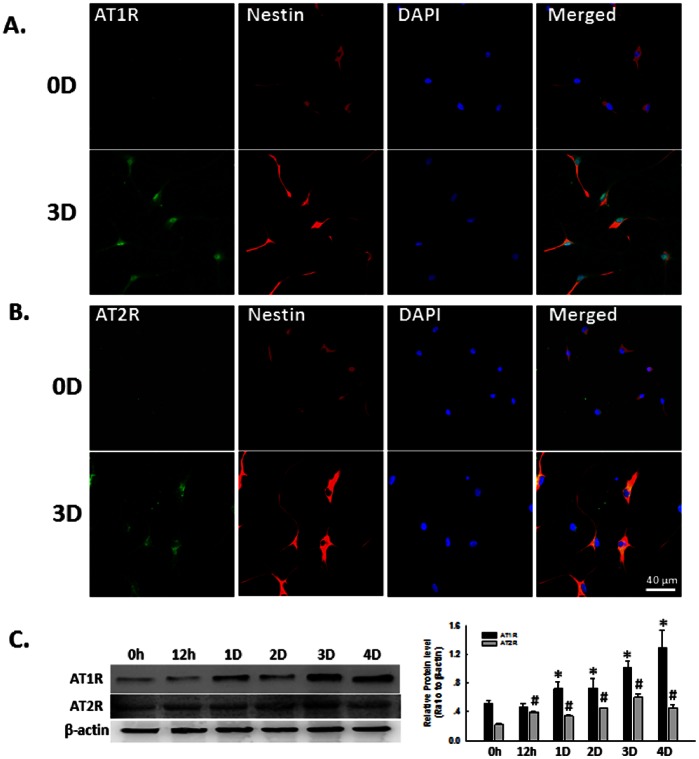
Expression pattern of AT1R and AT2R in NSCs. A) Representative immunofluorescence images showing the AT1R expression in NSCs. 0D: 0 day; 3D: 3 days. NSCs cultured at day 0 or day 3 were fixed with 4% paraformaldehyde followed by immunostaining for nestin (red), a neural stem cell marker, and AT1R (green). DAPI (blue): nuclei marker. Scale bar:40 µm. B) Representative immunofluorescence images showing the AT2R expression in NSCs. NSCs cultured at day 0 or day 3 were fixed with 4% paraformaldehyde followed by immunostaining for nestin (red) and AT2R (green). DAPI (blue): nuclei marker. Scale bar:40 µm. C) Representative blots (left panel) and mean data of relative blot density (right panel) showing expression pattern of AT1R and AT2R in NSCs. Expression of AT1R or AT2R was determined from NSCs cultured for 0 hour, 12 hours, 1 day, 2 days, 3 days and 4 days by Western Blot analysis. *P<0.05 *vs* AT1R expression in NSCs cultured at 0 hour; ^#^P<0.05 *vs* AT2R expression in NSCs cultured at 0 hour, n = 3 in each group.

Since Ang II mediates signaling via binding to its cognate receptor, AT1R and AT2R, we next examined the expression pattern of AT1R and AT2R in rat NSCs. Interestingly, we observed increased expression of both AT1R (Figure 2A) and AT2R (Figure 2B) in cultures from days 0 to day 3 by immunocytochemistry. These findings were further confirmed by Western-blot analysis (Figure 2C).

### Ang II-induced ERK and Akt Activation in NSCs

**Figure 3:**
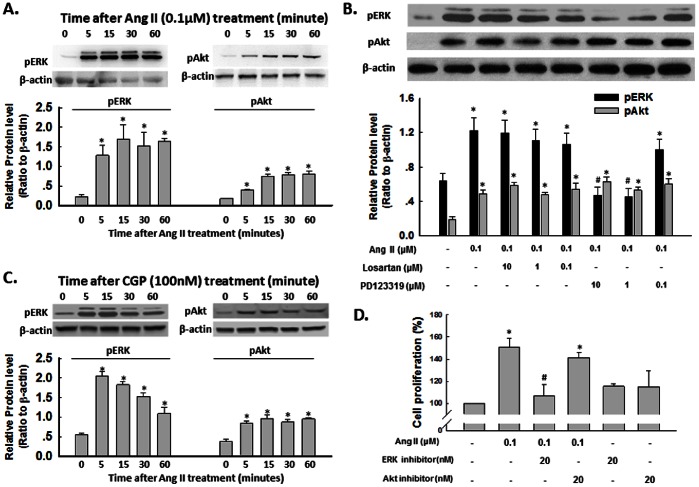
Ang II -induced ERK and Akt activation. A) Representative blots (upper panel) and mean data of relative blot density (lower panel) showing Ang II induced a rapid phosphorylation of ERK and Akt in NSCs. Cells were treated with Ang II for different time followed by western- blot analysis. *P<0.05 *vs* untreated group; n = 3 in each group. B) Representative blots (upper panel) and mean data of blot density (lower panel) showing blockade of AT1R and AT2R on phosphorylation of ERK and Akt. NSCs were pre-treated with specific antagonist to AT1R or AT2R for 1 hour followed by incubation with Ang II for 30 min. *P<0.05 *vs* untreated group, ^#^P<0.05 *vs* Ang II-treated group; n = 3 in each group. C) Representative blots (upper panel) and mean data of relative blot density (lower panel) showing CGP42112A induced a rapid phosphorylation of ERK and Akt in NSCs. Cells were treated with CGP42112A for different time followed by western-blot analysis. *P<0.05 *vs* untreated group; n = 3 in each group. D) Effect of blockade of ERK and Akt on Ang II-induced NSC proliferation. Cells were pretreated with specific ERK or Akt pathway inhibitor-U0126 or LY2940002 for 1 hour followed by treatment with Ang II for another 48 hours. Cell proliferation was assessed by CyQUANT Assay. *P<0.05 *vs* untreated group, ^#^P<0.05 *vs* Ang II- treated group; n = 5 in each group.

ERK/mitogen-activated protein kinase (MAPK) and PI3K/Akt pathway has been demonstrated to play a crucial role in cell proliferation [44], [45]. It was therefore of interest to examine the effect of Ang II on ERK and Akt regulation in rat NSCs. According to the results showing in Figure 2, we cultured NSCs for 4 days, and then performed the experiments. As shown in Figure 3A, exposure of NSCs to Ang II resulted in a sustained and time-dependent activation of ERK and Akt. However, only ERK, but not Akt, was blocked by pretreatment of cells with the AT2R antagonist-PD123319 for 1 hour, which significantly attenuated Ang II-mediated sustained activation of ERK (Fig. 3B). Consistent with the role of AT2R in NSC proliferation, AT2R agonist-CGP42112A exhibited the similar effect as Ang II, as shown in Figure 3C. Since MEK1/2 lies upstream of ERK, pretreatment of cells with MEK inhibitor U0126 resulted in abrogation of ERK phosphorylation induced by Ang II. So the functional role of Ang II-induced ERK activation in mediating NSC proliferation was corroborated using cell viability assay, wherein Ang II failed to exert its proliferation effect in cells pre-treated with MEK inhibitor-U0126, but not PI3K inhibitor-LY294002, thereby underscoring the role of this pathway in Ang II-mediated NSC proliferation (Fig. 3D).

### Ang II Decreased the Expression of K^+^ Channel in NSCs

**Figure 4:**
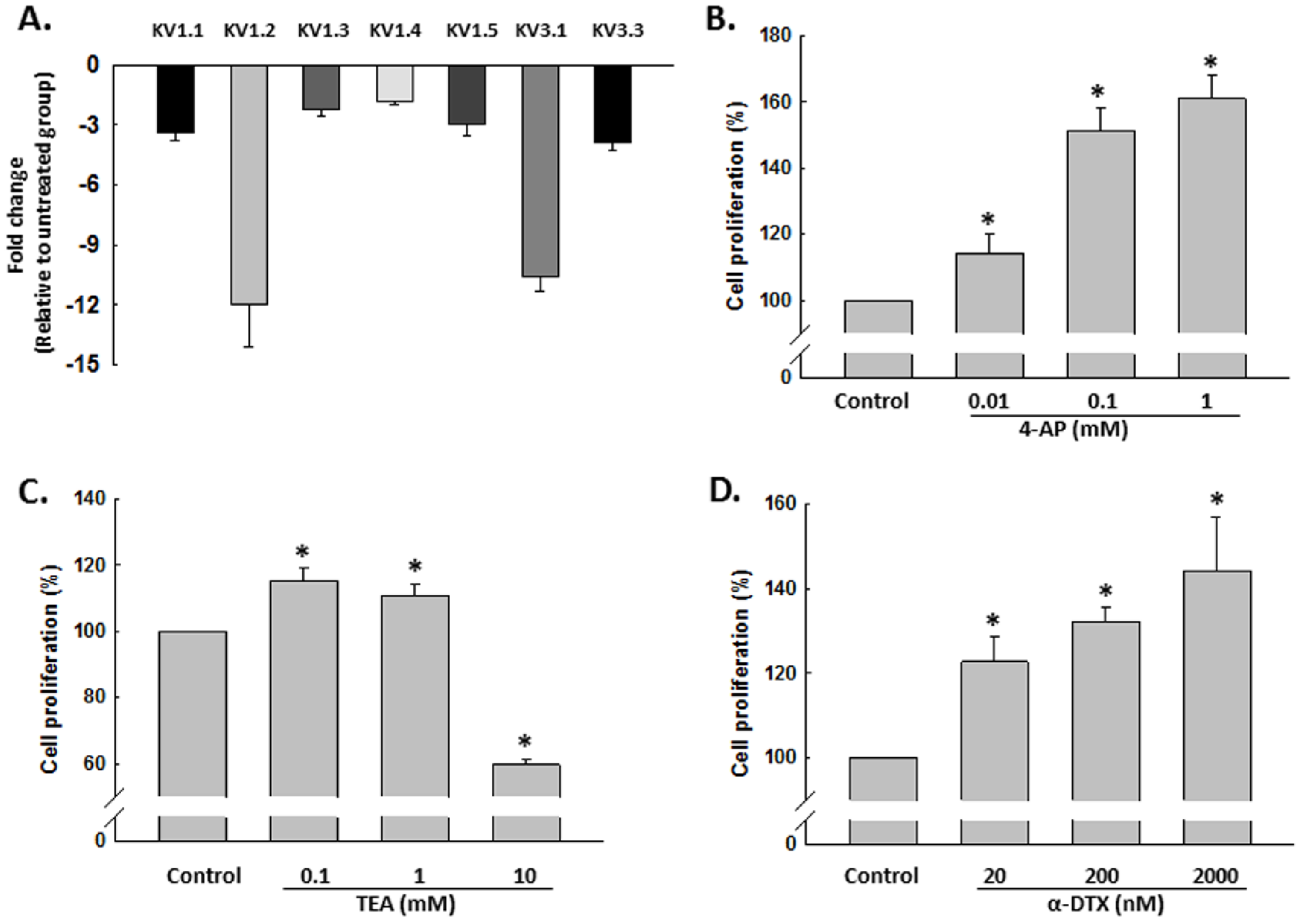
Ang II decreased the expression of K_v_ channel in NSCs. A) Effect of Ang II on the expression of different K_V_ channels by real-time RT-PCR assay. NSCs were treated with Ang II (0.1 µM) for 24 hours followed by RT-PCR. n = 3 in each group. B) K_V_ channel blockade with 4-AP induced NSC proliferation in concentration-dependent manner. NSCs were treated with different concentrations of 4-AP for 48 hours followed by CyQUANT assay. C) Effect of TEA on NSCs proliferation. NSCs were treated with different concentrations of TEA for 48 hours followed by CyQUANT assay. D) α-DTX induced NSC proliferation in concentration-dependent manner. NSCs were treated with different concentrations of α-DTX for 48 hours followed by CyQUANT assay. *P<0.05 *vs* untreated control group, n = 5 in each group.

Since cell proliferation can be regulated by modulation of K^+^ channel activity, Ang II has been shown to regulate both K^+^ current and K^+^ channel expression in different types of cell [36], [37]. It was reasonable to examine the effect of Ang II on K^+^ channel expression and K^+^ current in NSCs. As shown in Figure 4A, treatment of NSCs with Ang II (0.1 µM) for 24 hours specifically decreased mRNA level of tested K^+^ channel, in which K_V_1.2 and K_V_ 3.1 decreased more than 10 fold. Functional implication of K^+^ channel in NSC proliferation induced by Ang II was further corroborated using proliferation assays, wherein 4-AP (0.01, 0.1 and 1 mM), a specific K_V_ channel blocker, increased NSC proliferation in a concentration-dependent manner (Fig. 4B). These findings were further confirmed by K_V_1.2 and K_V_ 3.1 specific blocker, α-DTX and low dosage of TEA (0.1 mM) respectively, as shown in Figure 4C and 4D. These findings underpinned the critical role of K^+^ channel in regulation of NSC proliferation.

### Ang II-mediated Blockage of K^+^ Currents Lies Downstream of AT2R/ERK Pathway

**Figure 5:**
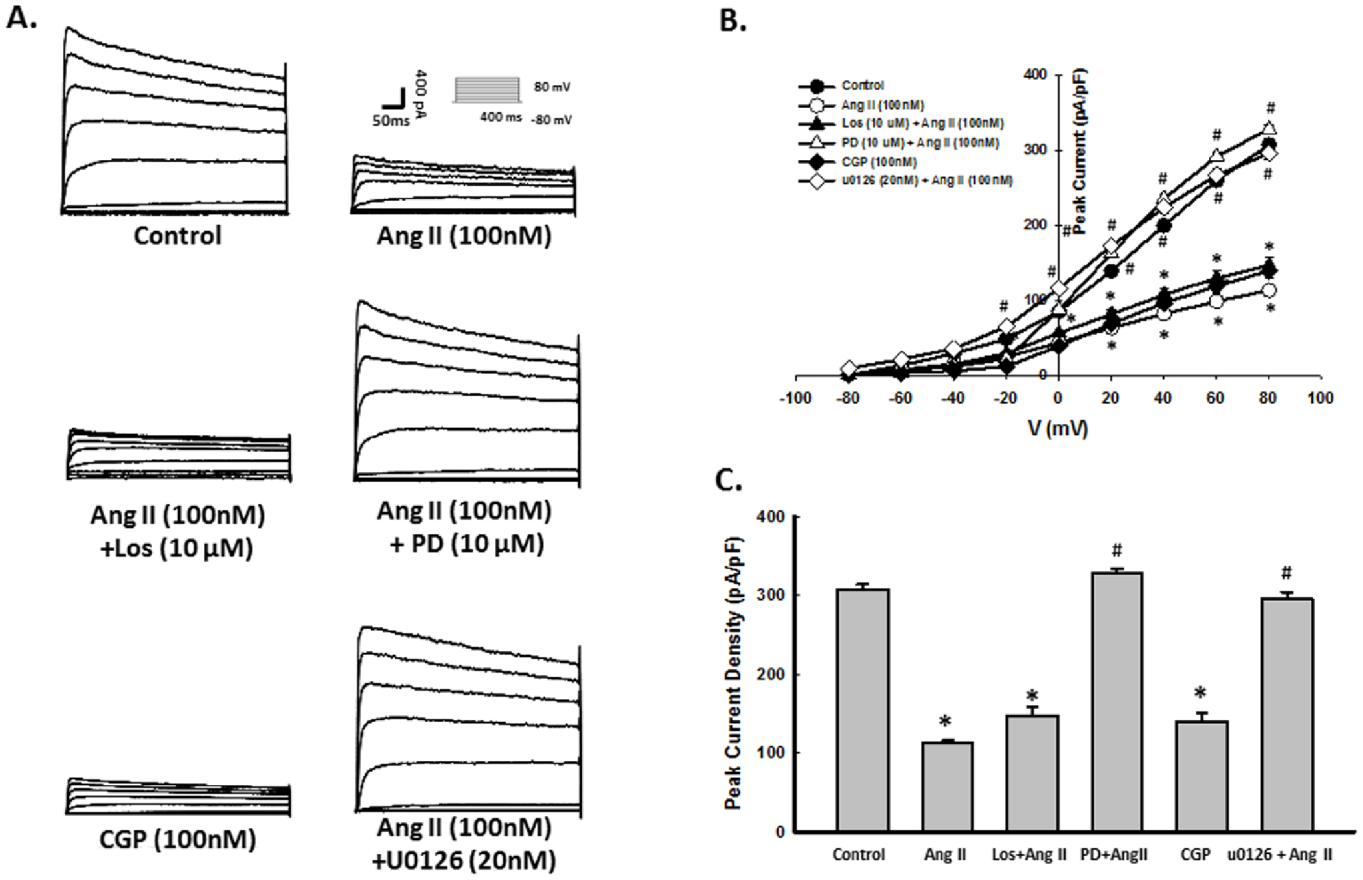
Ang II-mediated blockage of K^+^ currents lies downstream of AT2R/ERK pathway. A) Representative K^+^ current traces showing response of Ang II, Ang II+ PD123319, Ang II+Losartan, CGP42112A or Ang II+U0126 in NSCs evoked from −80 to 80 mV. NSCs were pre-treated with vehicle, Losartan, PD123319, or U0126 for 1 hour followed by incubation with Ang II or CGP42112A for 24 hours. B) Mean data of peak K^+^ current change from −80 to 80 mV. C) Peak K^+^ current change at +80 mV. *P<0.05 *vs* untreated control group, ^#^P<0.05 *vs* Ang II- treated group; n = 10 in each group.

Based on the premise that K_V_ channels mediate cell proliferation and that Ang II regulated K^+^ currents in other cell system, it was rationalized that K^+^ currents through AT2R is a pre-requisite for increased NSC proliferation mediated by Ang II. NSCs were treated with Ang II and CGP42112A for 48 hours, then subsequently assessed for K^+^ currents using whole-cell patch clamp. As shown in Figure 5A–C, Ang II and CGP42112A significantly decreased K^+^ currents in NSCs. The effect of Ang II on K^+^ currents was restored by pretreatment of PD123319, but not by losartan. These findings underpinned the role of AT2R in Ang II-mediated blockage of K^+^ currents. To further unravel the role of ERK pathway in Ang II-mediated blockage of K^+^ currents, NSCs were pretreated with the MEK inhibitor-U0126 and assessed for K^+^ currents. As shown in Figure 5A–C, pre-treatment of cells with U0126 markedly attenuated Ang II-induced blockage of K^+^ currents.

## Discussion

It is well-recognized that new dentate granule cells are continuously generated from NSCs and are integrated into the existing hippocampal circuitry in the adult mammalian brain through an orchestrated process termed adult neurogenesis [46]. Neurogenesis is regulated by a variety of physiological as well as pathological stimuli [12], [13], [14]. This study illuminates a novel prospective on the involvement of Ang II in regulation of NSC proliferation. Studies to determine whether Ang II treatment enhances the differentiation of NSCs into neurons or astrocytes are under investigation.

It is well-known that the cardiovascular and other actions of Ang II are mediated by AT1R and AT2R, which are seven transmembrane glycoproteins with 30% sequence similarity [2], [47], [48], [49], [50], [51], [52]. Mounting evidence have demonstrated that the functional local RAS have been found in such diverse organ system as the pancreas, heart, kidney as well as the nervous system [47], [48], [50], [51], [53]. Of note, in our NSC cell culture system, there is a time-dependent increase of AT1R and AT2R expression. Therefore, we speculate that RAS may be involved in NSC proliferation. As we expected, pretreatment of NSCs with AT2R antagonist inhibited Ang II-induced NSC proliferation. However, this finding was not consistent with the previous reports that AT1R activation enhanced the proliferation of somatic cells, such as smooth muscle cells and lung fibroblasts [16], [17], and DNA synthesis in mouse embryonic stem cells [54]. Thus, it is possible that distinct mechanisms underlie the effects of Ang II on proliferation of different types of stem cells. While the role of AT1R in NSC proliferation awaits for further investigation, the importance of the Ang II-AT2R axis in the maintenance of normal endocrine development has been suggested recently [55], which adds credence to our present findings.

In this study, we observed that Ang II induced ERK and Akt phosphorylation. These signaling pathways have been shown to regulate NSC proliferation [44], [45]. Interestingly, blocking AT2R resulted in suppression of Ang II-induced ERK activation, but not AT1R. The findings reported here on Ang II-mediated activation of ERK pathway are consistent with several lines of published reports [43], [56]. Consistent with the previous studies [22], [57], data presented here provide strong evidence that stimulation of AT2R induced activation of ERK/MAPK pathway.

Another interesting finding herein was the observation that inhibition of PI3K/Akt failed to reverse Ang II-mediated NSC proliferation thereby ruling out the potential role of this pathway. It is well known that activation of PI3K/Akt and its downstream pathway-NF-κB plays a key role in enhancing NSC proliferation following exposure to a wide array of stimuli [58], [59]. Our results are not consistent with previous reports that PI3K/Akt transduces intracellular signals that regulate adult NSC proliferation [60]. Interestingly, all of those studies use the NSCs from adult rats, in which, the expression of AT1R and AT2R pattern is total different from NSCs from fetal or neonate rats [8]. Thus, the different findings of involvement of PI3K/Akt in NSCs from adult and neonate animal indicate that Ang II and its receptor may play an important role in regulation of NSC proliferation.

Another novel finding of this study is the role of K^+^ channel in Ang II-mediated increased proliferation, thereby lending credence to previous reports indicating the involvement of K^+^ channels and K^+^ currents in cell proliferation [37]. Previous reports demonstrated the co-localization of K^+^ channel with the neural progenitor cells isolated from subventricular zone [61]. In our cell culture system, different subtypes of K_V_ channels were expressed in NSCs. Ang II is known to exert its action by regulation of K^+^ currents in neuronal cell system [62]. However, whether Ang II can regulate K^+^ currents in NSCs remains less clear. Herein we report that Ang II induced inhibition of K^+^ currents through activation of AT2R. Both AT1R and AT2R belong to the large family of G protein-coupled receptors with seven transmembrane domains [49], [50], [51], [52]. It is well established the mammalian K^+^ channels can be activated by G-protein-coupled receptors [63], [64]. Traditionally, Ang II was thought to inhibit K^+^ current via the activation of AT1R [38], [65] and stimulates K^+^ current via AT2R [40]. However, previous study also demonstrated the inhibition effect of Ang II on K^+^ current through AT2R [66]. Our findings provide another example of Ang II inhibit K^+^ currents via AT2R, but not AT1R. A key finding here is that Ang II-induced inhibition of K^+^ currents via ERK activation. It was evident from our findings that Ang II-mediated inhibition of K^+^ currents is ERK-dependent activation, and ensuing NSC proliferation. To further address the relevance of K_V_ channel in NSC proliferation, we test the effect of pan K_V_ channel blocker and specific K_V_ 1.2/K_V_ 3.1 blocker in NSC proliferation. 4-AP, α-DTX and low dosage of TEA significantly increased NSC proliferation, while high dosage of TEA inhibited NSC proliferation. It is reported that high concentration of TEA induced mammalian neuroblastoma cell swelling and decreased cell proliferation [67]. Whether same mechanism also involved in our system needs to be further investigated. Nevertheless, our findings are in agreement with the previous reports describing the involvement of K_V_ channel in NSC proliferation [35].

In summary, activation of the AngII/AT2R axis resulted in stimulation of ERK pathways leading to inhibition of K_V_ channels, which in turn, resulted in increased NSC proliferation. Taken together, our findings suggest that although the two pathways were upregulated by Ang II, only ERK was involved in Ang II-mediated increased proliferation of NSCs. A better understanding of these molecular pathways could be critical for the understanding how Ang II play important role in the NSC proliferation in the central nervous system.

## References

[1] Veerasingham SJ, Raizada MK (2003) Brain renin-angiotensin system dysfunction in hypertension: recent advances and perspectives. British journal of pharmacology 139: 191–202. 12770924 10.1038/sj.bjp.0705262 PMC1573858

[2] de Gasparo M, Catt KJ, Inagami T, Wright JW, Unger T (2000) International union of pharmacology. XXIII. The angiotensin II receptors. Pharmacological reviews 52: 415–472. 10977869

[3] Bumpus FM, Catt KJ, Chiu AT, DeGasparo M, Goodfriend T, et al (1991) Nomenclature for angiotensin receptors. A report of the Nomenclature Committee of the Council for High Blood Pressure Research. Hypertension 17: 720–721. 2022414 10.1161/01.hyp.17.5.720

[4] Mogi M, Horiuchi M (2013) Effect of angiotensin II type 2 receptor on stroke, cognitive impairment and neurodegenerative diseases. Geriatrics & gerontology international 13: 13–18. 22726823 10.1111/j.1447-0594.2012.00900.x

[5] Horiuchi M, Mogi M, Iwai M (2010) The angiotensin II type 2 receptor in the brain. Journal of the renin-angiotensin-aldosterone system : JRAAS 11: 1–6. 19861353 10.1177/1470320309347793

[6] Phillips MI, de Oliveira EM (2008) Brain renin angiotensin in disease. Journal of molecular medicine 86: 715–722. 18385968 10.1007/s00109-008-0331-5 PMC7095973

[7] Grady EF, Sechi LA, Griffin CA, Schambelan M, Kalinyak JE (1991) Expression of AT2 receptors in the developing rat fetus. The Journal of clinical investigation 88: 921–933. 1885777 10.1172/JCI115395 PMC295487

[8] Yu L, Zheng M, Wang W, Rozanski GJ, Zucker IH, et al (2010) Developmental changes in AT1 and AT2 receptor-protein expression in rats. J Renin Angiotensin Aldosterone Syst 11: 214–221. 20807798 10.1177/1470320310379065 PMC3035052

[9] Luo CX, Jin X, Cao CC, Zhu MM, Wang B, et al (2010) Bidirectional regulation of neurogenesis by neuronal nitric oxide synthase derived from neurons and neural stem cells. Stem cells 28: 2041–2052. 20845474 10.1002/stem.522

[10] Aimone JB, Wiles J, Gage FH (2006) Potential role for adult neurogenesis in the encoding of time in new memories. Nat Neurosci 9: 723–727. 16732202 10.1038/nn1707

[11] Kempermann G, Wiskott L, Gage FH (2004) Functional significance of adult neurogenesis. Curr Opin Neurobiol 14: 186–191. 15082323 10.1016/j.conb.2004.03.001

[12] Peng H, Whitney N, Wu Y, Tian C, Dou H, et al (2008) HIV-1-infected and/or immune-activated macrophage-secreted TNF-alpha affects human fetal cortical neural progenitor cell proliferation and differentiation. Glia 56: 903–916. 18383342 10.1002/glia.20665 PMC2644639

[13] Arvidsson A, Collin T, Kirik D, Kokaia Z, Lindvall O (2002) Neuronal replacement from endogenous precursors in the adult brain after stroke. Nature medicine 8: 963–970. 10.1038/nm747 12161747

[14] Nakatomi H, Kuriu T, Okabe S, Yamamoto S, Hatano O, et al (2002) Regeneration of hippocampal pyramidal neurons after ischemic brain injury by recruitment of endogenous neural progenitors. Cell 110: 429–441. 12202033 10.1016/s0092-8674(02)00862-0

[15] Rodgers KE, Xiong S, Steer R, diZerega GS (2000) Effect of angiotensin II on hematopoietic progenitor cell proliferation. Stem cells 18: 287–294. 10924095 10.1634/stemcells.18-4-287

[16] Wang Z, Rao PJ, Shillcutt SD, Newman WH (2005) Angiotensin II induces proliferation of human cerebral artery smooth muscle cells through a basic fibroblast growth factor (bFGF) dependent mechanism. Neuroscience letters 373: 38–41. 15555773 10.1016/j.neulet.2004.09.068

[17] Marshall RP, McAnulty RJ, Laurent GJ (2000) Angiotensin II is mitogenic for human lung fibroblasts via activation of the type 1 receptor. American journal of respiratory and critical care medicine 161: 1999–2004. 10852780 10.1164/ajrccm.161.6.9907004

[18] Meffert S, Stoll M, Steckelings UM, Bottari SP, Unger T (1996) The angiotensin II AT2 receptor inhibits proliferation and promotes differentiation in PC12W cells. Molecular and cellular endocrinology 122: 59–67. 8898348 10.1016/0303-7207(96)03873-7

[19] Stroth U, Meffert S, Gallinat S, Unger T (1998) Angiotensin II and NGF differentially influence microtubule proteins in PC12W cells: role of the AT2 receptor. Brain research Molecular brain research 53: 187–195. 9473667 10.1016/s0169-328x(97)00298-2

[20] Li JM, Mogi M, Tsukuda K, Tomochika H, Iwanami J, et al (2007) Angiotensin II-induced neural differentiation via angiotensin II type 2 (AT2) receptor-MMS2 cascade involving interaction between AT2 receptor-interacting protein and Src homology 2 domain-containing protein-tyrosine phosphatase 1. Molecular endocrinology 21: 499–511. 17068200 10.1210/me.2006-0005

[21] Laflamme L, Gasparo M, Gallo JM, Payet MD, Gallo-Payet N (1996) Angiotensin II induction of neurite outgrowth by AT2 receptors in NG108–15 cells. Effect counteracted by the AT1 receptors. The Journal of biological chemistry 271: 22729–22735. 8798447 10.1074/jbc.271.37.22729

[22] Gendron L, Laflamme L, Rivard N, Asselin C, Payet MD, et al (1999) Signals from the AT2 (angiotensin type 2) receptor of angiotensin II inhibit p21ras and activate MAPK (mitogen-activated protein kinase) to induce morphological neuronal differentiation in NG108–15 cells. Molecular endocrinology 13: 1615–1626. 10478850 10.1210/mend.13.9.0344

[23] Cao Z, Kelly DJ, Cox A, Casley D, Forbes JM, et al (2000) Angiotensin type 2 receptor is expressed in the adult rat kidney and promotes cellular proliferation and apoptosis. Kidney international 58: 2437–2451. 11115077 10.1046/j.1523-1755.2000.00427.x

[24] Clere N, Corre I, Faure S, Guihot AL, Vessieres E, et al (2010) Deficiency or blockade of angiotensin II type 2 receptor delays tumorigenesis by inhibiting malignant cell proliferation and angiogenesis. International journal of cancer Journal international du cancer 127: 2279–2291. 20143398 10.1002/ijc.25234

[25] Ager EI, Chong WW, Wen SW, Christophi C (2010) Targeting the angiotensin II type 2 receptor (AT2R) in colorectal liver metastases. Cancer cell international 10: 19. 20584290 10.1186/1475-2867-10-19 PMC2902462

[26] DeCoursey TE, Chandy KG, Gupta S, Cahalan MD (1984) Voltage-gated K+ channels in human T lymphocytes: a role in mitogenesis? Nature 307: 465–468. 6320007 10.1038/307465a0

[27] MacFarlane SN, Sontheimer H (2000) Changes in ion channel expression accompany cell cycle progression of spinal cord astrocytes. Glia 30: 39–48. 10696143 10.1002/(sici)1098-1136(200003)30:1<39::aid-glia5>3.0.co;2-s

[28] Wonderlin WF, Strobl JS (1996) Potassium channels, proliferation and G1 progression. The Journal of membrane biology 154: 91–107. 8929284 10.1007/s002329900135

[29] Pardo LA (2004) Voltage-gated potassium channels in cell proliferation. Physiology 19: 285–292. 15381757 10.1152/physiol.00011.2004

[30] Lang F, Shumilina E, Ritter M, Gulbins E, Vereninov A, et al (2006) Ion channels and cell volume in regulation of cell proliferation and apoptotic cell death. Contributions to nephrology 152: 142–160. 17065810 10.1159/000096321

[31] Li GR, Deng XL (2011) Functional ion channels in stem cells. World journal of stem cells 3: 19–24. 21607133 10.4252/wjsc.v3.i3.19 PMC3097936

[32] Attali B, Wang N, Kolot A, Sobko A, Cherepanov V, et al (1997) Characterization of delayed rectifier Kv channels in oligodendrocytes and progenitor cells. The Journal of neuroscience : the official journal of the Society for Neuroscience 17: 8234–8245. 9334399 10.1523/JNEUROSCI.17-21-08234.1997 PMC6573763

[33] Yasuda T, Bartlett PF, Adams DJ (2008) K(ir) and K(v) channels regulate electrical properties and proliferation of adult neural precursor cells. Molecular and cellular neurosciences 37: 284–297. 18023363 10.1016/j.mcn.2007.10.003

[34] Yasuda T, Adams DJ (2010) Physiological roles of ion channels in adult neural stem cells and their progeny. Journal of neurochemistry 114: 946–959. 20492359 10.1111/j.1471-4159.2010.06822.x

[35] Liebau S, Propper C, Bockers T, Lehmann-Horn F, Storch A, et al (2006) Selective blockage of Kv1.3 and Kv3.1 channels increases neural progenitor cell proliferation. J Neurochem 99: 426–437. 17029597 10.1111/j.1471-4159.2006.03967.x

[36] Gao L, Li Y, Schultz HD, Wang WZ, Wang W, et al (2010) Downregulated Kv4.3 expression in the RVLM as a potential mechanism for sympathoexcitation in rats with chronic heart failure. Am J Physiol Heart Circ Physiol 298: H945–955. 20044444 10.1152/ajpheart.00145.2009 PMC2838543

[37] Wang SP, Wang JA, Luo RH, Cui WY, Wang H (2008) Potassium channel currents in rat mesenchymal stem cells and their possible roles in cell proliferation. Clinical and experimental pharmacology & physiology 35: 1077–1084. 18505444 10.1111/j.1440-1681.2008.04964.x

[38] Sumners C, Zhu M, Gelband CH, Posner P (1996) Angiotensin II type 1 receptor modulation of neuronal K+ and Ca2+ currents: intracellular mechanisms. The American journal of physiology 271: C154–163. 8760041 10.1152/ajpcell.1996.271.1.C154

[39] Zhu M, Neubig RR, Wade SM, Posner P, Gelband CH, et al (1997) Modulation of K+ and Ca2+ currents in cultured neurons by an angiotensin II type 1a receptor peptide. The American journal of physiology 273: C1040–1048. 9316425 10.1152/ajpcell.1997.273.3.C1040

[40] Kang J, Sumners C, Posner P (1993) Angiotensin II type 2 receptor-modulated changes in potassium currents in cultured neurons. The American journal of physiology 265: C607–616. 8214016 10.1152/ajpcell.1993.265.3.C607

[41] Caballero R, Gomez R, Moreno I, Nunez L, Gonzalez T, et al (2004) Interaction of angiotensin II with the angiotensin type 2 receptor inhibits the cardiac transient outward potassium current. Cardiovascular research 62: 86–95. 15023555 10.1016/j.cardiores.2003.12.029

[42] Tian C, Murrin LC, Zheng JC (2009) Mitochondrial fragmentation is involved in methamphetamine-induced cell death in rat hippocampal neural progenitor cells. PloS one 4: e5546. 19436752 10.1371/journal.pone.0005546 PMC2677674

[43] Li HW, Gao YX, Matsuura T, Martynyuk A, Raizada MK, et al (2005) Adenoviral-mediated neuron specific transduction of angiotensin II type 2 receptors. Regulatory peptides 126: 213–222. 15664669 10.1016/j.regpep.2004.10.005

[44] Peltier J, O’Neill A, Schaffer DV (2007) PI3K/Akt and CREB regulate adult neural hippocampal progenitor proliferation and differentiation. Developmental neurobiology 67: 1348–1361. 17638387 10.1002/dneu.20506

[45] Learish RD, Bruss MD, Haak-Frendscho M (2000) Inhibition of mitogen-activated protein kinase kinase blocks proliferation of neural progenitor cells. Brain research Developmental brain research 122: 97–109. 10915910 10.1016/s0165-3806(00)00064-x

[46] Venkatesan A, Nath A, Ming GL, Song H (2007) Adult hippocampal neurogenesis: regulation by HIV and drugs of abuse. Cell Mol Life Sci 64: 2120–2132. 17530169 10.1007/s00018-007-7063-5 PMC11138422

[47] Wright JW, Harding JW (2011) Brain renin-angiotensin–a new look at an old system. Progress in neurobiology 95: 49–67. 21777652 10.1016/j.pneurobio.2011.07.001

[48] Wright JW, Harding JW (2013) The brain renin-angiotensin system: a diversity of functions and implications for CNS diseases. Pflugers Archiv : European journal of physiology 465: 133–151. 22535332 10.1007/s00424-012-1102-2

[49] Porrello ER, Delbridge LM, Thomas WG (2009) The angiotensin II type 2 (AT2) receptor: an enigmatic seven transmembrane receptor. Frontiers in bioscience : a journal and virtual library 14: 958–972. 10.2741/3289 19273111

[50] Steckelings UM, Rompe F, Kaschina E, Namsolleck P, Grzesiak A, et al (2010) The past, present and future of angiotensin II type 2 receptor stimulation. Journal of the renin-angiotensin-aldosterone system : JRAAS 11: 67–73. 19861348 10.1177/1470320309347791

[51] Guimond MO, Gallo-Payet N (2012) The Angiotensin II Type 2 Receptor in Brain Functions: An Update. International journal of hypertension 2012: 351758. 23320146 10.1155/2012/351758 PMC3540774

[52] Guimond MO, Gallo-Payet N (2012) How does angiotensin AT(2) receptor activation help neuronal differentiation and improve neuronal pathological situations? Frontiers in endocrinology 3: 164. 23267346 10.3389/fendo.2012.00164 PMC3525946

[53] Paul M, Poyan Mehr A, Kreutz R (2006) Physiology of local renin-angiotensin systems. Physiological reviews 86: 747–803. 16816138 10.1152/physrev.00036.2005

[54] Han HJ, Han JY, Heo JS, Lee SH, Lee MY, et al (2007) ANG II-stimulated DNA synthesis is mediated by ANG II receptor-dependent Ca(2+)/PKC as well as EGF receptor-dependent PI3K/Akt/mTOR/p70S6K1 signal pathways in mouse embryonic stem cells. Journal of cellular physiology 211: 618–629. 17219409 10.1002/jcp.20967

[55] Leung KK, Liang J, Ma MT, Leung PS (2012) Angiotensin II type 2 receptor is critical for the development of human fetal pancreatic progenitor cells into islet-like cell clusters and their potential for transplantation. Stem cells 30: 525–536. 22162314 10.1002/stem.1008

[56] Huang XC, Richards EM, Sumners C (1996) Mitogen-activated protein kinases in rat brain neuronal cultures are activated by angiotensin II type 1 receptors and inhibited by angiotensin II type 2 receptors. The Journal of biological chemistry 271: 15635–15641. 8663175 10.1074/jbc.271.26.15635

[57] Stroth U, Blume A, Mielke K, Unger T (2000) Angiotensin AT(2) receptor stimulates ERK1 and ERK2 in quiescent but inhibits ERK in NGF-stimulated PC12W cells. Brain research Molecular brain research 78: 175–180. 10891597 10.1016/s0169-328x(00)00093-0

[58] Kaus A, Widera D, Kassmer S, Peter J, Zaenker K, et al (2010) Neural stem cells adopt tumorigenic properties by constitutively activated NF-kappaB and subsequent VEGF up-regulation. Stem Cells Dev 19: 999–1015. 20184430 10.1089/scd.2009.0416

[59] Piotrowska MJ, Widera D, Kaltschmidt B, an der Heiden U, Kaltschmidt C (2006) Mathematical model for NF-kappaB-driven proliferation of adult neural stem cells. Cell Prolif 39: 441–455. 17109630 10.1111/j.1365-2184.2006.00403.x PMC6495974

[60] Peltier J, O’Neill A, Schaffer DV (2007) PI3K/Akt and CREB regulate adult neural hippocampal progenitor proliferation and differentiation. Dev Neurobiol 67: 1348–1361. 17638387 10.1002/dneu.20506

[61] Smith DO, Rosenheimer JL, Kalil RE (2008) Delayed rectifier and A-type potassium channels associated with Kv 2.1 and Kv 4.3 expression in embryonic rat neural progenitor cells. PloS one 3: e1604. 18270591 10.1371/journal.pone.0001604 PMC2225502

[62] Pan SJ, Zhu M, Raizada MK, Sumners C, Gelband CH (2001) ANG II-mediated inhibition of neuronal delayed rectifier K+ current: role of protein kinase C-alpha. American journal of physiology Cell physiology 281: C17–23. 11401823 10.1152/ajpcell.2001.281.1.C17

[63] Clapham DE (1994) Direct G protein activation of ion channels? Annual review of neuroscience 17: 441–464. 10.1146/annurev.ne.17.030194.002301 8210183

[64] Lober RM, Pereira MA, Lambert NA (2006) Rapid activation of inwardly rectifying potassium channels by immobile G-protein-coupled receptors. The Journal of neuroscience : the official journal of the Society for Neuroscience 26: 12602–12608. 17135422 10.1523/JNEUROSCI.4020-06.2006 PMC6674890

[65] Gelband CH, Zhu M, Lu D, Reagan LP, Fluharty SJ, et al (1997) Functional interactions between neuronal AT1 and AT2 receptors. Endocrinology 138: 2195–2198. 9112419 10.1210/endo.138.5.5236

[66] Chu KY, Cheng Q, Chen C, Au LS, Seto SW, et al (2010) Angiotensin II exerts glucose-dependent effects on Kv currents in mouse pancreatic beta-cells via angiotensin II type 2 receptors. American journal of physiology Cell physiology 298: C313–323. 19889960 10.1152/ajpcell.00575.2008

[67] Rouzaire-Dubois B, Dubois JM (1998) K+ channel block-induced mammalian neuroblastoma cell swelling: a possible mechanism to influence proliferation. The Journal of physiology 510 (Pt 1): 93–102. 10.1111/j.1469-7793.1998.093bz.x PMC2231012 9625869

